# Path Planning in Localization Uncertaining Environment Based on Dijkstra Method

**DOI:** 10.3389/fnbot.2022.821991

**Published:** 2022-03-11

**Authors:** Can Wang, Chensheng Cheng, Dianyu Yang, Guang Pan, Feihu Zhang

**Affiliations:** School of Marine Science and Technology, Northwestern Polytechnical University, Xi'an, China

**Keywords:** path planning, greedy search, cumulative error estimation, global planning, Dijkstra

## Abstract

Path planning obtains the trajectory from one point to another with the robot's kinematics model and environment understanding. However, as the localization uncertainty through the odometry sensors is inevitably affected, the position of the moving path will deviate further and further compared to the original path, which leads to path drift in GPS denied environments. This article proposes a novel path planning algorithm based on Dijkstra to address such issues. By combining statistical characteristics of localization error caused by dead-reckoning, the replanned path with minimum cumulative error is generated with uniforming distribution in the searching space. The simulation verifies the effectiveness of the proposed algorithm. In a real scenario with measurement noise, the results of the proposed algorithm effectively reduce cumulative error compared to the results of the conventional planning algorithm.

## 1. Introduction

To obtain the optimal trajectory from one point to another, path planning needs to combine the robot's geometric and dynamic information (Bidot et al., 2013), environment map (Peng and Green, 2019), the initial state and target state (Choset et al., 2005), etc. According to task requirements, the optimal path seeks the shortest length and the best energy (Ibraheem and Hassan Ajeil, 2018). In specific tasks, path planning is commonly performed by combining sensor type and performance, carrier kinematics and dynamics characteristics, and task requirements.

Classical path planning methods consists of heuristic searching, sampling planning, and model-dependent methods (Yilmaz et al., 2008). In particular, when localizing through IMU (Tick et al., 2013), visual odometry (He et al., 2020), or other sensors (Paull et al., 2014), none of the mentioned methods considers the localization uncertainty issue. However, the GPS may be subject to some limitations in practical applications, especially in underwater scenarios (Li et al., 2019). In applications with GPS-denied, it is not feasible to combine the robot's motion attributes with inaccurate odometry sensors, which will cause localization errors in long-term missions. It is generally accepted that positioning errors do not affect the planning task since planning is first performed and then control decisions are made. In robot tasks where errors exist, however, it is also possible to impact localization errors by changing the path planning strategy.

To address the cumulative error of navigation, many studies first perform accurate statistical analysis on it. Miller et al. (2010) proposed an error state formula for the navigation algorithm of an underwater vehicle. The kinematics model of the system is augmented with unknown parameters from the sensor model, and the difference between the estimation of the real augmented system equation is expressed as the error state system. And a Kalman filter is designed to estimate this error state by the measurement residuals of the auxiliary sensor. Yin et al. (2013) established a strap-down inertial navigation system error model based on various error sources of inertial components. By using Particle Swarm Optimization (PSO) to optimize the parameters of SVM, the positioning error prediction method of a navigation system is realized. By redesigning the system parameters and using data recalculation algorithms, Xu et al. (2014) proposed an improved alignment method for Strapdown Inertial Navigation System (SINS) based on Doppler Velocity Log (DVL). Dai et al. (2016) proposes a particle swarm algorithm to identify the error parameters of the Delta parallel robot, and the geometric parameter errors can be identified by a simple iterative process. Mansouri et al. (2020) settles positioning uncertainty by defining adaptive weights for tracking position and speed reference points, and calculating based on the uncertainty associated with measurement. Accurate error estimation methods facilitate the correction and compensation of navigation positioning. Nevertheless, few studies have effectively integrated error estimation with the navigation planning process, which is uncharted territory.

To effectively reduce the navigation error, generally intermittent global position correction methods based on GPS, SLAM, acoustic positioning, (Thomson et al., 2017; Chew and Zakaria, 2019; Marchel et al., 2020), etc. There are also studies on error compensation based on artificial intelligence methods (Brossard et al., 2020) or combined with the kinematics of the robot (Batista et al., 2013). However, in the planning stage, the navigation error cannot be effectively reduced without a determined path. Therefore, the existing research generally solves such problems through fault-tolerant planning. Carlson et al. (2013) proposed and compared three different strategies for estimating the change of the robot's motion, which effectively reduced the probability of collisions and avoided sources of error in industrial scenarios. Eaton et al. (2017) proposed a robust *Partially Observable Markov Decision Process (POMDP)* formula, which provides the capability of planning and tracking with limited observations. Lv et al. (2019) cited the dense connection method to improve the Q-networks structure to solve the issue of robot drift by adopting the framework of a dense network. Sainte Catherine and Lucet (2020) combined with the improved *Hybrid Reciprocating Speed Obstacle (HRVO)* method of tracking error estimation, and adapting the speed obstacle paradigm to agents with dynamic constraints and unreliable velocity estimation. Yilmaz et al. (2021) uses the fuzzy logic network to model dynamic uncertainty, and proposes a new definition of the error-like vector containing the pseudo-inverse of the Jacobian matrix. The current method only considers the fault-tolerance of path planning and does not apply the mechanism of the cumulative error to avoid tracking drift, i.e., does not consider the impact of the motion after planning.

By considering the perceptual uncertainty, some planning methods consider the generation and elimination strategies of planned path errors, and thus, new planning methods are designed. Pilania and Gupta (2017) designs sensor measurements that depend only on the samples, achieving higher uncertainty reduction by placing more samples in regions with higher uncertainty reduction while maintaining enough samples in regions with poor uncertainty reduction. It also uses uncertainty measures (instead of distance) to connect new samples to neighboring nodes, achieving an efficient and high-quality planning capability. Park et al. (2018) achieved collision avoidance path planning by considering the uncertainty of the time-varying trajectories of linearly increasing Autonomous Ground Vehicles (AGVs) and obstacles, modeling the error covariance using a tracking filter designed to estimate motion information, and employing a probabilistic approach to calculate the collision risk combined with the dynamic characteristics of AGVs. Papachristos et al. (2019) designed a paradigm that follows a hierarchical optimization objective and executes it in a backward horizon manner to implement an uncertainty-aware path planning strategy. Combining adaptive error sampling for generating possible path candidates with a utility-based approach, Lee et al. (2020) implements a path planning task for safe parking under perceptible uncertainty, which takes into account detection errors and makes optimal decisions under uncertainty. Uncertainty generation is mainly obtained through passive sensors, and unfortunately, the current capability to rely on inertial navigation alone for path planning under uncertainty needs to be further explored.

However, in practical applications, system errors and deviations are inevitable with sensor registration problems. Failure to use the control strategy to optimize the planning and motion process, a disastrous deviation will occur in the tracking process. Our previous study (Wang et al., 2021) applies reinforcement learning to address this issue and obtains a path with a relatively smaller cumulative error by generating a probability sampling. As the limitation of sampling, the global optimal solution cannot be obtained.

This article combines a qualitative and quantitative analysis of the ranging error and traversal advantage of the greedy search algorithm in the path planning process. To minimize the accumulated errors in navigation, we obtain an ideal path that can achieve high accuracy tracking. The key innovation is the theoretical modeling from the systematic perspective of error estimation and planning based on greedy search in a practical scene. In scenarios where measurement errors exist, the proposed algorithm is effective in reducing the path error concerning the underlying Dijkstra method. To the best of the author's knowledge, this is the first study that considers the cumulative error of tracking in the pre-planning process and performs global corrections to form paths with minimal cumulative error.

The main contributions of this article are as follows:

- Through the statistical qualitative and quantitative analysis of the cumulative error by odometry positioning, the qualitative and quantitative expressions for path planning are summarized.
- Improve the map exploration method of Dijkstra to adapt to the qualitative expression of reducting cumulative error.
- By iterating and optimizing the cumulative errors of the paths, the results of their statistics and the global optimal path are obtained.

This article is organized as follows: The second part analyzes the mathematical representation and statistical characteristics of the cumulation error. The third part proposes a path planning framework based on the improved Dijkstra method and optimized cumulative error. In the fourth part, the simulation planning results are compared and analyzed, and the results are discussed. Finally, the fifth part concludes the full article and discusses possible directions for future study.

## 2. Methodological Background

When the global positioning system is unavailable, the robot has to utilize the attitude sensor and inertial sensor (gyro and accelerometer) to perform dead-reckoning. Assuming odometry sensor measurement is only presented in polar coordinates, and the corresponding noises are distributed with *Independent Identically Distribution (IID)*, which is determined based on the comparative statistics of the measured value and the true value in Fallon et al. (2010). As the presence of noise, robot positioning by heading projection will produce a continuous accumulation of errors. Hence, the robot has to calibrate its positions regularly.

The main challenge of numerical analysis of errors is the drift caused by relative noise measurements, i.e., the cumulative error increases nonlinearly with distance or time. This article uses statistical properties to study the growth rate of cumulative error in our previous article (Zhang et al., 2013). In this article, the robot is viewed as a mass, i.e., there are no kinematic constraints. This means that localization information can only be derived from inertial navigation measurements and cannot be corrected for localization based on kinematic models. Still, the proposed method applies to all types of robots, since it only considers planning paths and does not involve path tracking strategies.

**Figure 1:**
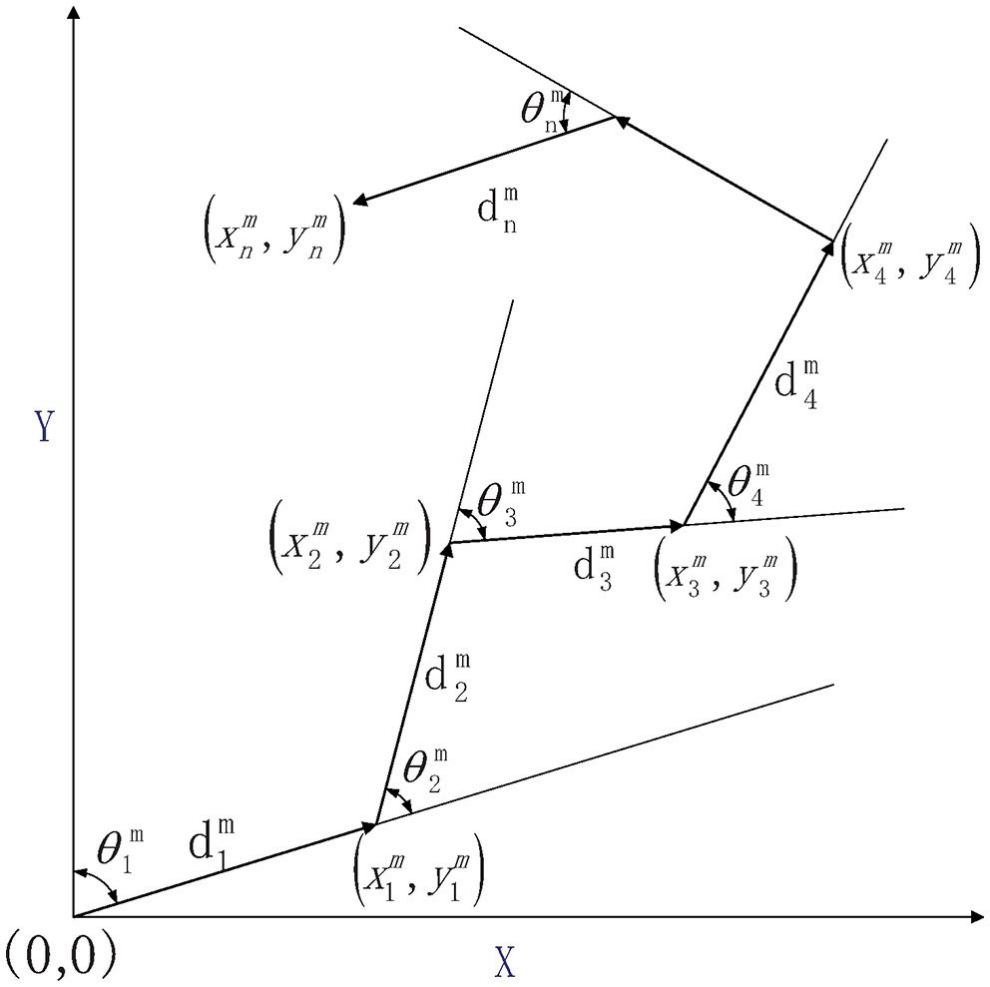
Relationship between robot relative measurement and position.

The robot position is estimated based on angle and distance in polar coordinates, as shown in Figure 1. Define the corresponding metric:


(1) $$\begin{array}{r} \theta_{n}^{m}={\bar{\theta }}_{n}+{\tilde{\theta }}_{n};d_{n}^{m}={\bar{d}}_{n}+{\tilde{d}}_{n} \end{array}$$

where *n* is the time index, *d* and θ represents relative distance and direction between consecutive frames. The pose measurement $\left(\theta_{n}^{m},\;d_{n}^{m}\right)$ is then consisted of ground truth $\left(\bar{\theta_{n}},\;\bar{d_{n}}\right)$ and error $\left(\tilde{\theta_{n}},\;\tilde{d_{n}}\right)$ with SD δ_θ_ and δ_*d*_.

The principle of dead-reckoning in the Cartesian coordinate system is as follows:


(2) $$\begin{array}{r} x_{n}^{m}=\sum_{i=1}^{n}\left(d_{i}^{m}\sin\sum_{j=1}^{i}\theta_{j}^{m}\right) \end{array}$$

(3) $$\begin{array}{r} y_{n}^{m}=\sum_{i=1}^{n}\left(d_{i}^{m}\cos\sum_{j=1}^{i}\theta_{j}^{m}\right) \end{array}$$

The accumulation of drift by noise measurement is unbounded. The lower bound can be estimated by Cramer2Rao bound (Arrichiello et al., 2012), but the upper bound cannot be estimated by traditional methods, especially when there are no basic facts. However, the error distribution properties of multiple statistics can be used for the statistical estimation of errors.

When the true value is known, the trajectory can also be expressed as:


(4) $$\begin{array}{l} x_{n}^{m}=\bar{x_{n}}+{\tilde{x}}_{n}\\ \;=\sum_{i=1}^{n}\left(d_{i}^{m}\sin\sum_{j=1}^{i}\theta_{j}^{m}\right)\\ \;=\sum_{i=1}^{n}\left(\left(\bar{d_{i}}+{\tilde{d}}_{i}\right)\sin\sum_{j=1}^{i}\left(\bar{\theta_{j}}+{\tilde{\theta }}_{j}\right)\right)\\ \text{ =}\left(\sum_{i=1}^{n}\bar{d_{i}}+\sum_{i=1}^{n}{\tilde{d}}_{i}\right)\cdot [\sin\sum_{j=1}^{i}\bar{\theta_{j}}\cos\sum_{j=1}^{i}{\tilde{\theta }}_{j}\\ \;+\cos\sum_{j=1}^{i}\bar{\theta_{j}}\sin\sum_{j=1}^{i}{\tilde{\theta }}_{j}] \end{array}$$

Then the mathematical expression of the cumulative error in the *x*-direction can be obtained:


(5) $$\begin{array}{r} \begin{array}{c} \tilde{x_{n}}=\sum_{i=1}^{n}\bar{d_{i}}[\begin{array}{c} \sin\sum_{j=1}^{i}\bar{\theta_{j}}\left(\cos\sum_{j=1}^{i}\tilde{\theta_{j}}-1\right)\\ +\cos\sum_{j=1}^{i}\bar{\theta_{j}}\sin\sum_{j=1}^{i}\tilde{\theta_{j}} \end{array}]\\ \;+\sum_{i=1}^{n}\tilde{d_{i}}[\begin{array}{c} \sin\sum_{j=1}^{i}\bar{\theta_{j}}\cos\sum_{j=1}^{i}\tilde{\theta_{j}}\\ +\cos\sum_{j=1}^{i}\bar{\theta_{j}}\sin\sum_{j=1}^{i}\tilde{\theta_{j}} \end{array}] \end{array} \end{array}$$

In fact, the cumulative error depends to a large extent on basic facts. In addition, the expected and variance of the cumulative error are estimated based on statistical properties:


(6) $$\begin{array}{r} E\left[\tilde{x}|\bar{\theta },\bar{d}\right]=\sum_{i=1}^{n}\bar{d_{i}}\left[\sin\sum_{j=1}^{i}\bar{\theta_{j}}\left(e^{-\frac{i\delta_{\theta }^{2}}{2}-1}\right)\right] \end{array}$$

(7) $$\begin{array}{r} \begin{array}{c} \text{var}\left(\tilde{x}|\bar{\theta },\bar{d}\right)=E\left[\tilde{x}|\bar{\theta },\bar{d}\right]-E^{2}\left[\tilde{x}|\bar{\theta },\bar{d}\right]\\ \;=A+B+C-E^{2}\left[\tilde{x}|\bar{\theta },\bar{d}\right] \end{array} \end{array}$$

where:


(8) $$\begin{array}{l} A=\sum_{i=1}^{n}\bar{d_{i}^{2}}[\sin^{2}\sum_{j=1}^{i}\bar{\theta_{j}}\left(0.5e^{-2i\delta_{\theta }^{2}}+1.5-2e^{-\frac{i\delta_{\theta }^{2}}{2}}\right)\\ \;+0.5\cos^{2}\sum_{j=1}^{i}\bar{\theta_{j}}\left(e^{-2i\delta_{\theta }^{2}}+1\right)] \end{array}$$

(9) $$\begin{array}{l} B=2\sum_{i=1}^{n-1}\sum_{p=1+i}^{n}\bar{d_{i}}\bar{d_{p}}\\ \;\left\{\begin{array}{l} \sin^{2}\sum_{j=1}^{i}\bar{\theta_{j}}\cos\Delta \bar{\theta }\left[\begin{array}{l} 1+0.5\left(1+e^{-2i\delta_{\theta }^{2}}\right)e^{-0.5\left(p-i\right)\delta_{\theta }^{2}}\\ e^{-0.5i\delta_{\theta }^{2}}-e^{-0.5i\delta_{\theta }^{2}}e^{-0.5\left(p-i\right)\delta_{\theta }^{2}} \end{array}\right]\\ +\sin\sum_{j=1}^{i}\bar{\theta_{j}}\sin\Delta \bar{\theta }\cos\sum_{j=1}^{i}\bar{\theta_{j}}\\ \left[\begin{array}{l} 1+0.5\left(1+e^{-2i\delta_{\theta }^{2}}\right)e^{-0.5\left(p-i\right)\delta_{\theta }^{2}}+1\\ -e^{-0.5i\delta_{\theta }^{2}}-e^{-0.5i\delta_{\theta }^{2}}e^{-0.5\left(p-i\right)\delta_{\theta }^{2}}\\ -0.5\left(1-e^{-2i\delta_{\theta }^{2}}\right)e^{-0.5\left(p-i\right)\delta_{\theta }^{2}} \end{array}\right]\\ +\cos^{2}\sum_{j=1}^{i}\bar{\theta_{j}}\cos\Delta \bar{\theta }\cdot 0.5\left(1-e^{-2i\delta_{\theta }^{2}}\right)e^{-0.5\left(p-i\right)\delta_{\theta }^{2}} \end{array}\right\} \end{array}$$

(10) $$\begin{array}{l} C=\sum_{i=1}^{n}[0.5\sin^{2}\sum_{j=1}^{i}\bar{\theta_{j}}\left(e^{-2i\delta_{\theta }^{2}}+1\right)\\ \;+0.5\cos^{2}\sum_{j=1}^{i}\bar{\theta_{j}}\left(1-e^{-2i\delta_{\theta }^{2}}\right)] \end{array}$$

The above formula is an explicit expression of expectation and variance of cumulative error. Since the global planning map is a priori, this article uses the true value to calculate expectation and variance. However, the ground truth is quite challenging to acquire in real scenarios. To effectively evaluate the error in a real scene, the expected values of the true moment are evaluated conditional on the noisy relative measurements:


(11) $$\begin{array}{r} E\left[\tilde{x_{n}^{m}}\right]=\sum_{i=1}^{n}d_{i}^{m}\left(e^{-i\delta_{\theta }^{2}}-e^{-0.5i\delta_{\theta }^{2}}\right)\sin\sum_{j=1}^{i}\theta_{j}^{m} \end{array}$$

(12) $$\begin{array}{r} \text{var}\left(\tilde{x_{n}^{m}}\right)=A_{1}+B_{1}+C_{1}-E^{2}\left[\tilde{x_{n}^{m}}\right] \end{array}$$

where:


(13) $$\begin{array}{r} \begin{array}{c} A_{1}=\sum_{i=1}^{n}{\left(d_{i}^{m}\right)}^{2}\left\{\begin{array}{c} \left(\begin{array}{c} 0.5e^{-2i\delta_{\theta }^{2}}+1.5\\ -2e^{-0.5i\delta_{\theta }^{2}} \end{array}\right)\\ \left[\begin{array}{c} 0.5\left(1+e^{-2i\delta_{\theta }^{2}}\right)\sin^{2}\sum_{j=1}^{i}\theta_{j}^{m}\\ +0.5\left(1-e^{-2i\delta_{\theta }^{2}}\right)\cos^{2}\sum_{j=1}^{i}\theta_{j}^{m} \end{array}\right]\\ +0.5\left(1+e^{-2i\delta_{\theta }^{2}}\right)\\ \left[\begin{array}{c} 0.5\left(1+e^{-2i\delta_{\theta }^{2}}\right)\cos^{2}\sum_{j=1}^{i}\theta_{j}^{m}\\ +0.5\left(1+e^{-2i\delta_{\theta }^{2}}\right)\sin^{2}\sum_{j=1}^{i}\theta_{j}^{m} \end{array}\right] \end{array}\right\} \end{array} \end{array}$$

(14) $$\begin{array}{l} B_{1}=2\sum_{i=1}^{n-1}\sum_{p=1+i}^{n}d_{i}^{m}d_{p}^{m}\\ \;\left\{\begin{array}{l} \left[\begin{array}{l} 0.5\left(1+e^{-2i\delta_{\theta }^{2}}\right)\cdot \sin^{2}\sum_{j=1}^{i}\theta_{j}^{m}\\ +0.5\left(1-e^{-2i\delta_{\theta }^{2}}\right)\cdot \cos^{2}\sum_{j=1}^{i}\theta_{j}^{m} \end{array}\right]\\ \cdot \left[\cos\Delta \theta^{m}e^{-0.5\left(p-i\right)\delta_{\theta }^{2}}\right]\left[\cdots \right]\\ +[\sin\sum_{j=1}^{i}\theta_{j}^{m}\cdot \sin\Delta \theta^{m}\cdot \cos\sum_{j=1}^{i}\theta_{j}^{m}\\ \cdot e^{-2i\delta_{\theta }^{2}}e^{-0.5\left(p-i\right)\delta_{\theta }^{2}}]\left[\cdots \right]\\ +\left[\begin{array}{l} 0.5\left(1+e^{-2i\delta_{\theta }^{2}}\right)\cdot \cos^{2}\sum_{j=1}^{i}\theta_{j}^{m}\\ +0.5\left(1-e^{-2i\delta_{\theta }^{2}}\right)\cdot \sin^{2}\sum_{j=1}^{i}\theta_{j}^{m} \end{array}\right]\\ \left[\cos\Delta \theta^{m}e^{-0.5\left(p-i\right)\delta_{\theta }^{2}}\right]\left[\cdots \right] \end{array}\right\} \end{array}$$

(15) $$\begin{array}{r} C_{1}=\sum_{i=1}^{n}\left\{\begin{array}{c} 0.25\left(e^{-2i\delta_{\theta }^{2}}+1\right)\\ \left[\begin{array}{c} \left(e^{-2i\delta_{\theta }^{2}}+1\right)\cdot \sin^{2}\sum_{j=1}^{i}\theta_{j}^{m}\\ +\left(1-e^{-2i\delta_{\theta }^{2}}\right)\cos^{2}\sum_{j=1}^{i}\theta_{j}^{m} \end{array}\right]\\ +0.25\left(1-e^{-2i\delta_{\theta }^{2}}\right)\\ \left[\begin{array}{c} \left(1+e^{-2i\delta_{\theta }^{2}}\right)\cdot \cos^{2}\sum_{j=1}^{i}\theta_{j}^{m}\\ +\left(1-e^{-2i\delta_{\theta }^{2}}\right)\cdot \sin^{2}\sum_{j=1}^{i}\theta_{j}^{m} \end{array}\right] \end{array}\right\} \end{array}$$

More details could be found in Zhang and Knoll (2016) in the same manner, the complete cumulative errors are, therefore, calculated. In the next section, the Dijkstra-based global exploration method will first be used to traverse the map and determine the error-minimizing path for each location by evaluating the error of each path, thus achieving the task of reducing path drift.

## 3. Path Planning Method Based on Dijkstra

To obtain a globally optimal path with the smallest error in the prior map, it is necessary to traverse the entire map and generate an error map. That is, similar to the “breadcrumbs map,” the error map has nothing to do with the endpoint but only with the starting point. Meanwhile, a greedy algorithm means that only the locally optimal solution is selected, but the part relative to the starting point is known, which is conducive to the optimization of the algorithm. Therefore, this algorithm can only choose the global traversal method, not the heuristic method. This article improved the Dijkstra algorithm based on its principle and the qualitative results of error statistical calculations. At the same time, the quantitative calculation of path error is applied to iterate and update the error map, and finally, obtain the global error map. In the case of a given endpoint, the minimum error path can be quickly obtained through the global error map.

### 3.1. Improved Dijkstra

Breadth-First Search (BFS) (Broder et al., 2000) or Dijkstra (Kang et al., 2008) can be used to traverse the map, which is suitable for obtaining a global error map. However, the calculation of the cumulative error needs to be based on the entire path rather than part of the path segment, which does not apply to algorithms based on father-node exploration. Therefore, based on the results of Section II and previous study, the cumulative error has a great relationship with angle change of path measurement. That is, relative to starting point, the later the robot changes its angle, the smaller the cumulative error of path. The improved Dijkstra method will make the path generation of each point based on the latest turn path of starting point in the process of traversing the global map.

Since the Dijkstra algorithm is graph-based, we first initialize graph ***G*** and give a starting point. This algorithm is not to obtain the shortest path but to obtain a path that turns farther from starting point based on the nature of the cumulative error. The algorithm needs to initialize an empty set ***S*** to store those vertices that have been traversed and initialize a set ***Q*** which includes all vertices ***G***.*V*. ***Q*** uses the data structure of the smallest priority queue, in which the key is the number of angle changes from starting point to vertex, expressed as *trun*_*num*. Additionally, the vertex with the least number of angle changes is popped up each time.

In the rasterized map, the change of the robot's movement angle is discrete. This article chose {(0, 1), (1, 1), (1, 0), (1, −1), (0, −1), (−1, −1), (−1, 0), (−1, 1)} as optional movement directions ***G***.*Adj*[*u*]. To reduce the cumulative error of each path, we limit the angle change of each vertex adjacent point, i.e., the angle of each movement $|\theta |\leq \frac{\pi }{4}$. In other words, ***G***.*Adj*_*limited*[*u*] has only 3 adjacent vertices.

For the weight of the edge, we first make the path go straight, and have to make a turn before turning. In the algorithm, ω_*d*(*u, v*) is the distance from *u*→*v*, and ω(*u, v, u*.π) is the number of turns of the edge *u*→*v*. Since the traversal only considers the current node and adjacent nodes, the father node of the current node *u*.π is also required. The final algorithm will first traverse the nodes that have not turned, and then traverse the paths with fewer turns until the initial global error graph is generated. The complete pseudo-code of the improved Dijkstra algorithm is shown in Algorithm 1. The algorithm aims to facilitate subsequent point set updates and calculations by generating large error variances.

**Algorithm 1:** Improved Dijkstra.

**Require:** Dijkstra(***G***, *s*)
**Ensure:** Original global error map ***G***_*error*
1: INITIALIZE-SINGLE-SOURCE(***G***, *s*)
2: **for** each node *n* ∈ ***G***.*V* **do**
3: *n*.*turn*_*num* = 0
4: *n*.*d* = ∞
5: *n*.π = *NIL*
6: **end for**
7: *s*.*turn*_*num* = 0
8: ***S*** = ∅
9: ***Q*** = ***G***.*V*
10: **while** ***Q*** ≠ ∅ **do**
11: *u*= heappop (***Q***)
12: ***S*** = ***S*** ∪ {*u*}
13: **for** each node *v* ∈ ***G***.*Adj*_*limited*[*u*] **do**
14: **if** *v*.*turn*_*num* > *u*.*turn*_*num* + ω(*u, v, u*.π) **then**
15: *v*.*turn*_*num* = *u*.*turn*_*num* + ω(*u, v, u*.π)
16: *v*.π = *u*
17: *v*.*d* = *u*.*d* + ω_*d*(*u, v*)
18: *v*.*error*= Equation (6)
19: *v*.*path* = *PATH*(*s, v*)
20: **end if**
21: **end for**
22: **end while**

In the scenario where the sensor exists errors, the statistics of the cumulative error of the entire path are simple, but the error in the planning stage cannot be measured. Similar to the inability to obtain the best-first search strategy for the shortest path in concave obstacle environments, in addition, common planning methods are unable to move toward minimum error from the beginning. This is since larger errors may occur in the following trips, leading to larger overall deviations. This article evaluates the cumulative error based on the entire path from starting point to each point. Since the global map is a priori, the cumulative error of each path could be calculated through the true value of each measurement $\left(\bar{\theta },\;\bar{d}\right)$ based on Equation (6).

### 3.2. Global Iteration Strategy

In the initial global error map, the path from starting point will pass through obstacles and intersect. That is, some points will be reached by the paths on both sides of the obstacle together, which results in different cumulative error values for this point. For the points where there are differences in cumulative error caused by different paths, this article initializes and updates the minimum priority queue ***Q***_*dif* to determine the point set that needs to be iteratively calculated. In the iterative process, the error of the point set is recalculated and the path is updated to obtain a path with a smaller cumulative error for each point. The pseudo-code of strategy for updating queue is shown in Algorithm 2.

**Algorithm 2:** Update Iteration Point Set.

**Require:** Original global error map ***G***_*error*
**Ensure:** Point set to be iterated ***Q***_*dif*
1: ***Q***_*dif* = ∅
2: ***Q***_*temp* = ***G***.*V*
3: **while** ***Q***_*temp* ≠ ∅ **do**
4: *u*= heappop (***Q***_*temp*)
5: **for** each node *v* ∈ ***G***.*Adj*[*u*] **do**
6: **if** *v*.*value* > *A* **or** *v*.*value*_*absolute* > *B* **then**
7: ***Q***_*dif* = ***Q***_*dif*∪{*v*}
8: **end if**
9: **end for**
10: **end while**

To accurately get each point that needs to be iterated, we need to update the key-value *value* of each point in the traversal map in advance. In this article, it is defined as: *v*.*value* = max(***G***.*Adj*[*u*].*error* − *v*.*error*)/*v*.*d*, which is due to the smaller scale of the map and the absolute difference in cumulative error is not obvious. For maps with obvious error differences, we can judge whether the point needs iteration according to absolute error difference *v*.*value*_*absolute* = max(***G***.*Adj*[*u*].*error* − *v*.*error*). To adapt to different scale maps, we simultaneously apply two benchmarks to update the queue ***Q***_*dif*.

For point set ***Q***_*dif*, the algorithm traverses its adjacent nodes each time to calculate the minimum error. The algorithm requires that error difference is caused by the path passing through two sides of an obstacle, so the correlation between the current node path and adjacent node path needs to be calculated. To be logical, we define *CORRELATION*(*path*1, *path*2) = ∑(*dis*(*path*1, *path*2) < *D*)/*LEN*(*path*). It should be noted that, as the short distance between adjacent nodes, if their paths pass on the same side of an obstacle, the path correlation will be close to 1. The complete pseudo-code of the iteration strategy is shown in Algorithm 3.

**Algorithm 3:** Error Map Iteration Strategy.

**Require:** Point set to be iterated ***Q***_*dif*
**Ensure:** Final error map ***G***_*error*
1: **while** ***Q***_*dif* ≠ ∅ **do**
2: *u*= heappop (***Q***_*dif*)
3: **for** each node *v* ∈ ***G***.*Adj*[*u*] **do**
4: **if** *ERROR*(*v*.*path* + *u*) < *u*.*error* **and** *CORRELATION*(*v*.*path* + *u, u*.*path*) < *C* **then**
5: *u*.π = *v*
6: *u*.*path* = *v*.*path* + *u*
7: *u*.*error* = *ERROR*(*v*.*path* + *u*)
8: **end if**
9: **end for**
10: **end while**

In the algorithm, *A, B, C*, and *D* each represent a threshold constant, which is only for adjusting the algorithm effect and has no other representative meaning. Better convergence can be achieved by dynamically setting the threshold size according to the map size and task requirements.

## 4. Simulation

### 4.1. Implementation Details

**Figure 2:**
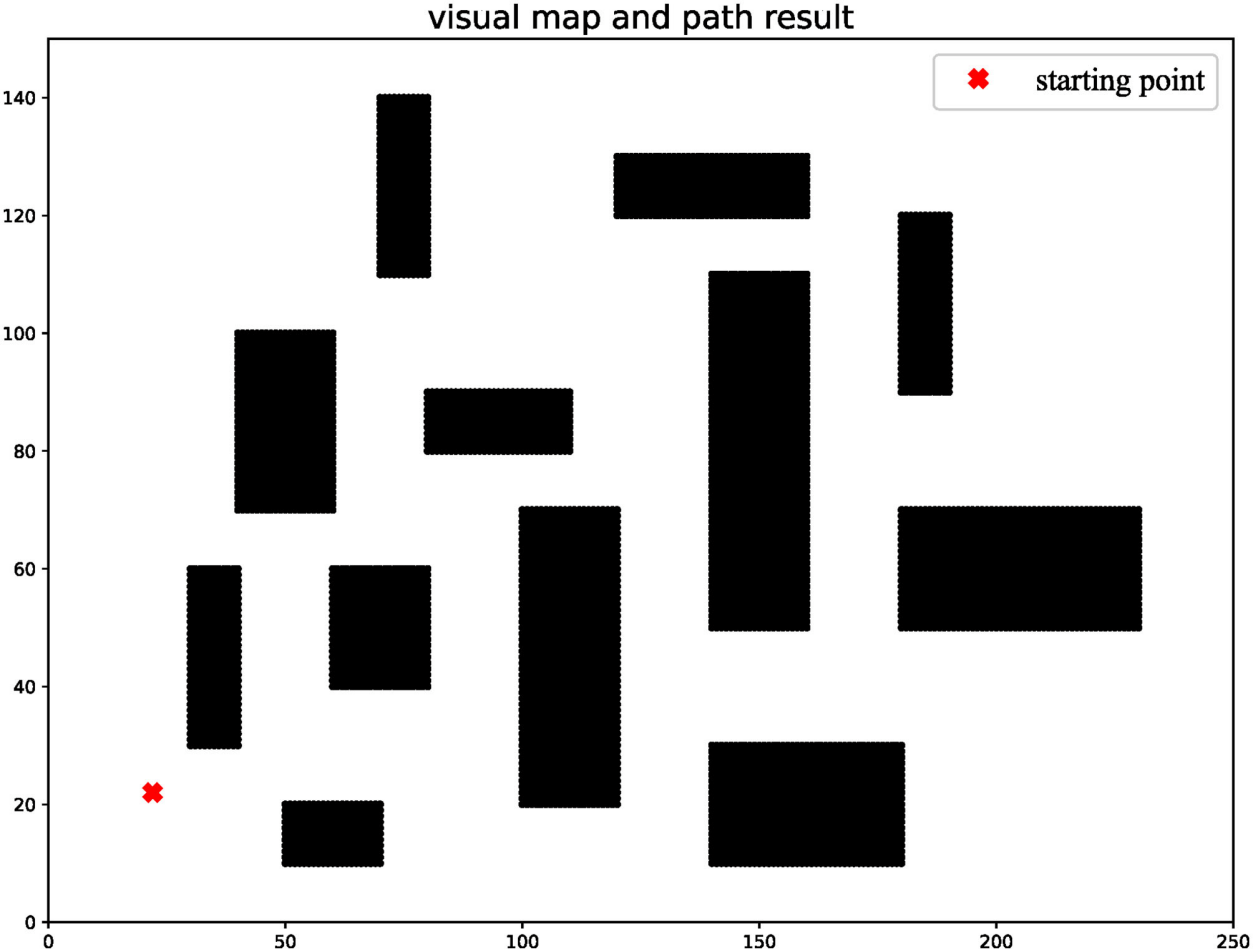
Initial global map.

A 2D grid map is used as a graphical basis for algorithmic simulations. In this article, a map with a scale of 250/150 was chosen and the starting point was randomly set to (22, 22). The priority map consists of obstacles, driveable areas, and boundaries as shown in Figure 2. In general, the robot can accurately reach the end-point through the tracking process, with the help of high-precision GPS. Considering cumulative error generated by the noise-ranging sensor when GPS-denied, this article assumed that sensor error satisfies the Gaussian distribution, i.e., the error distributions in distance and angle are *N*(0, 0.01) and *N*(0, 0.02).

**Figure 3:**
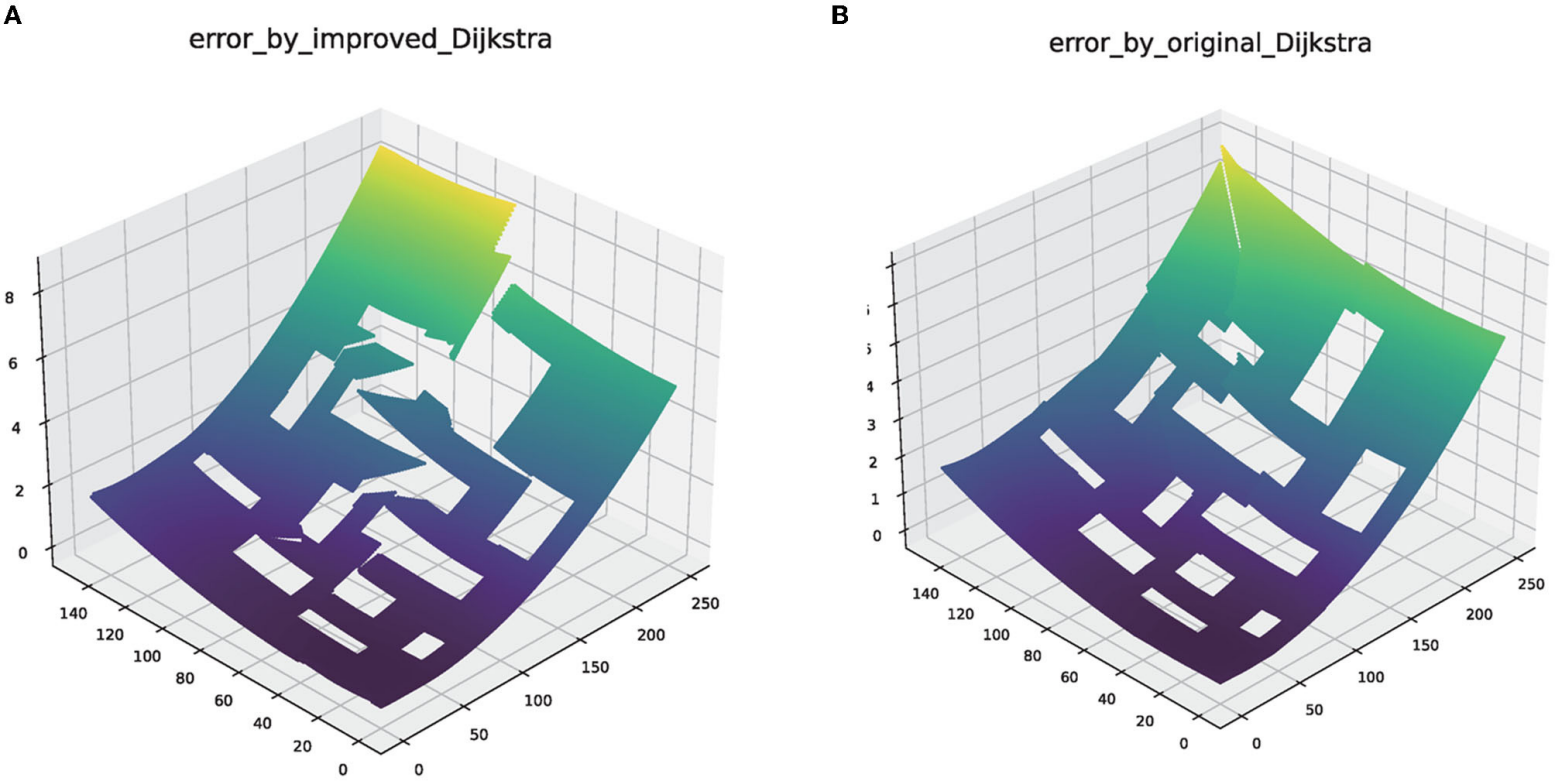
The error map generated by the original Dijkstra and improved Dijkstra. **(A)** The error map of original Dijkstra. **(B)** The error map of original Dijkstra.

The original Dijkstra method can only find the shortest path, which is not the path with the smallest cumulative error in individual scenarios. Especially every time robots pass an obstacle, it will cause a fault in the error map. The improved Dijkstra can delay turns from the starting point to each point, which is achieved by turning restrictions. The initial path graph generated by improved Dijkstra is conducive to the realization of later iterative convergence. The improved error graph is shown in Figure 3.

**Figure 4:**
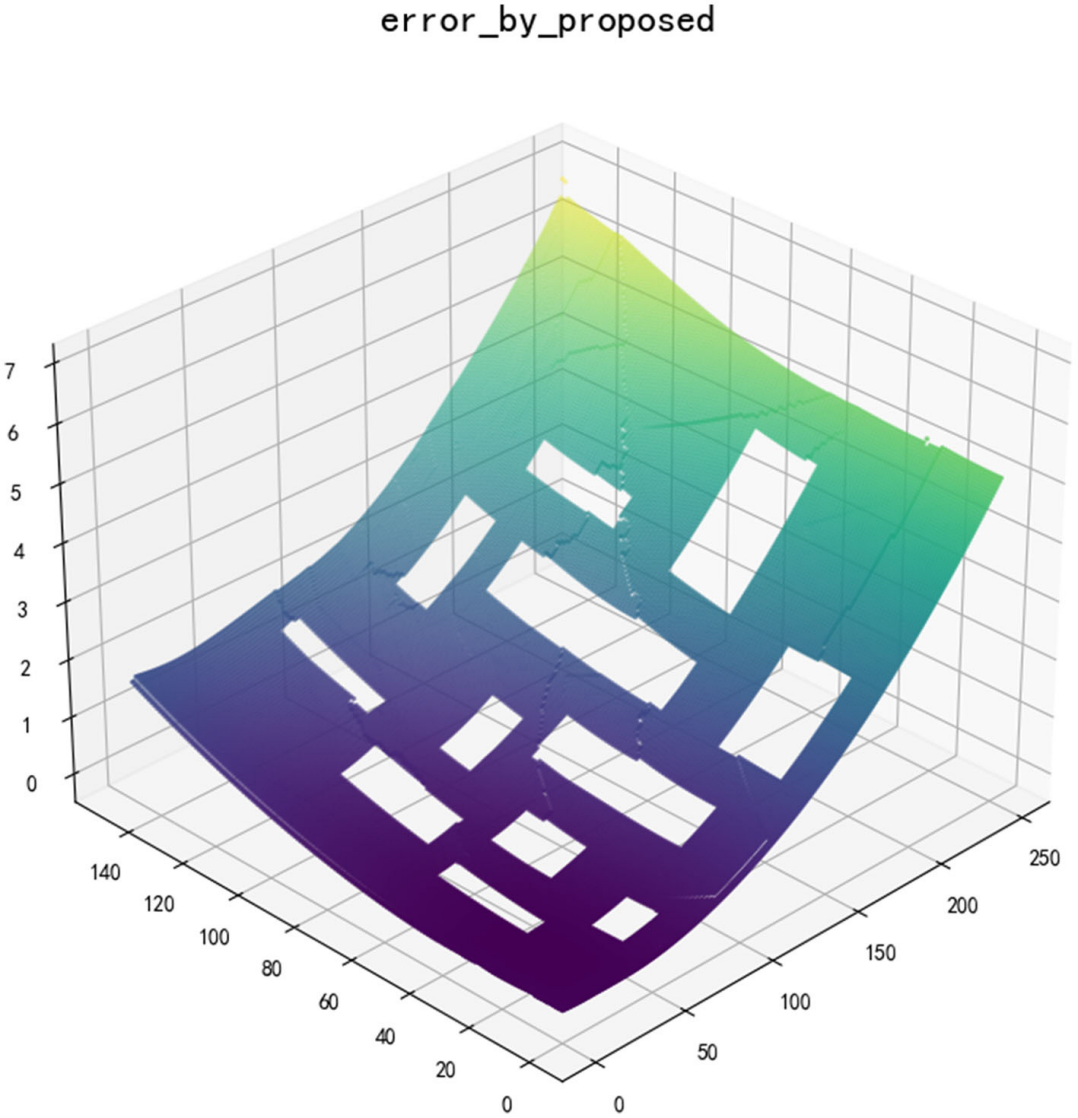
Global error map after iteration.

It is necessary to set a reasonable threshold in the update point set and algorithm iteration. According to the map scale in this article, we set the minimum allowable *value* that is *A* = 0.001, the path absolute error difference *B* = 0.1, the minimum allowable correlation between two paths *C* = 0.8, and the path correlation judgment distance is based on *D* = 5.0. After multiple iterations of the algorithm, the smallest error global map is finally generated, as shown in Figure 4. Additionally, the path of each point in the error map can be obtained by the way of parent node search, namely *PATH*(*s, u*).

**Figure 5:**
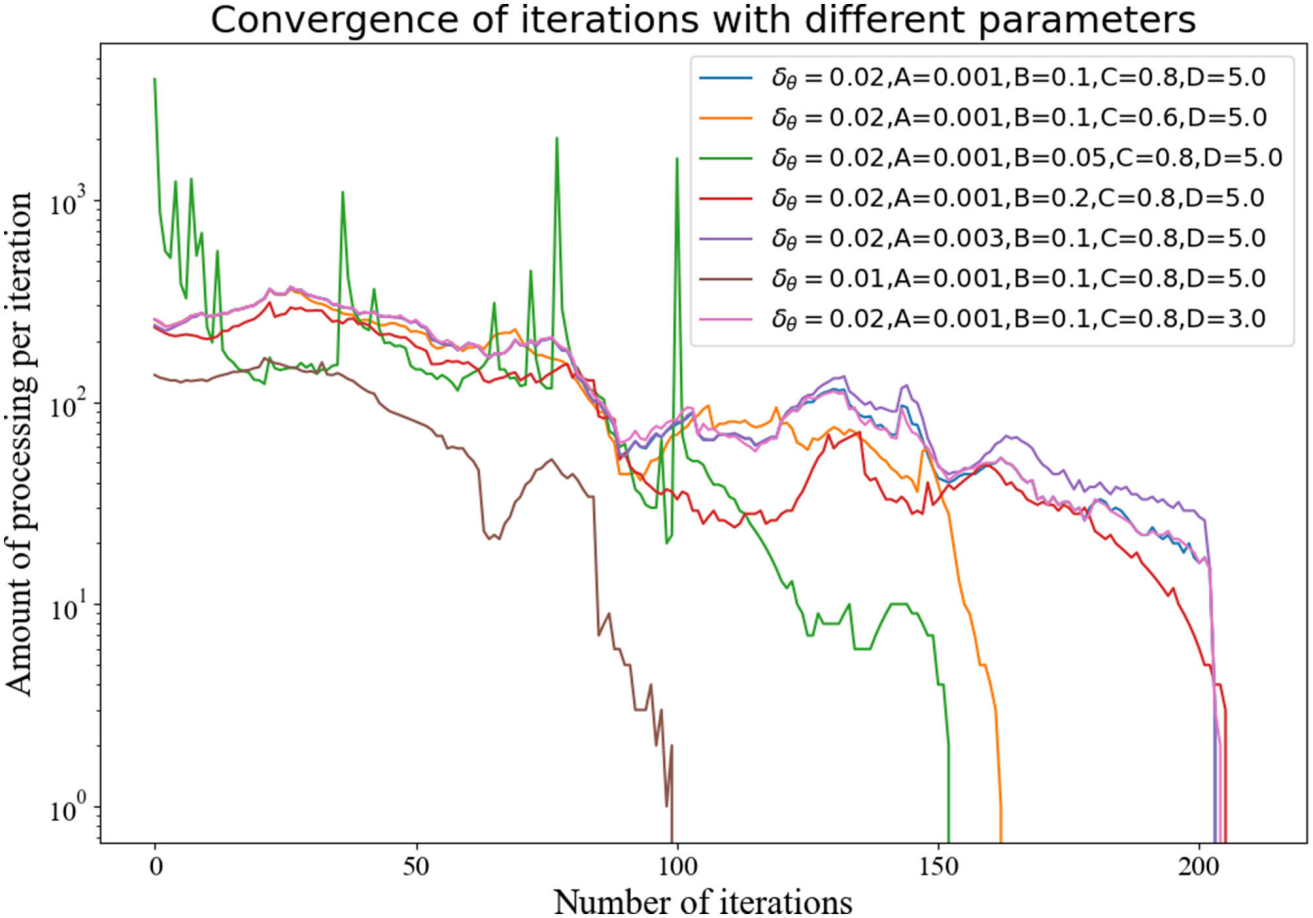
The number of calculations per iteration under different parameters.

For different thresholds, there are some differences in the convergence ability of the algorithm, although convergence results can be obtained for all. Additionally, this algorithm is also suitable for sensor calculation with different error distributions. We changed the settings of the relevant values, and the number of points processed in each iteration eventually tended to 0, as shown in Figure 5.

### 4.2. Analysis

Using the classic path planning algorithm, all points in drivable areas can be reached from starting point through at least one path. However, considering the influence of cumulative error caused by the above-mentioned sensor noise, the actual path will deviate from the original path and endpoint to a large extent. By adding measurement noise, we select a few representative path results and apply Equation (11) calculation to compare the effect of the planning algorithm in this article to reduce cumulative error.

**Figure 6:**
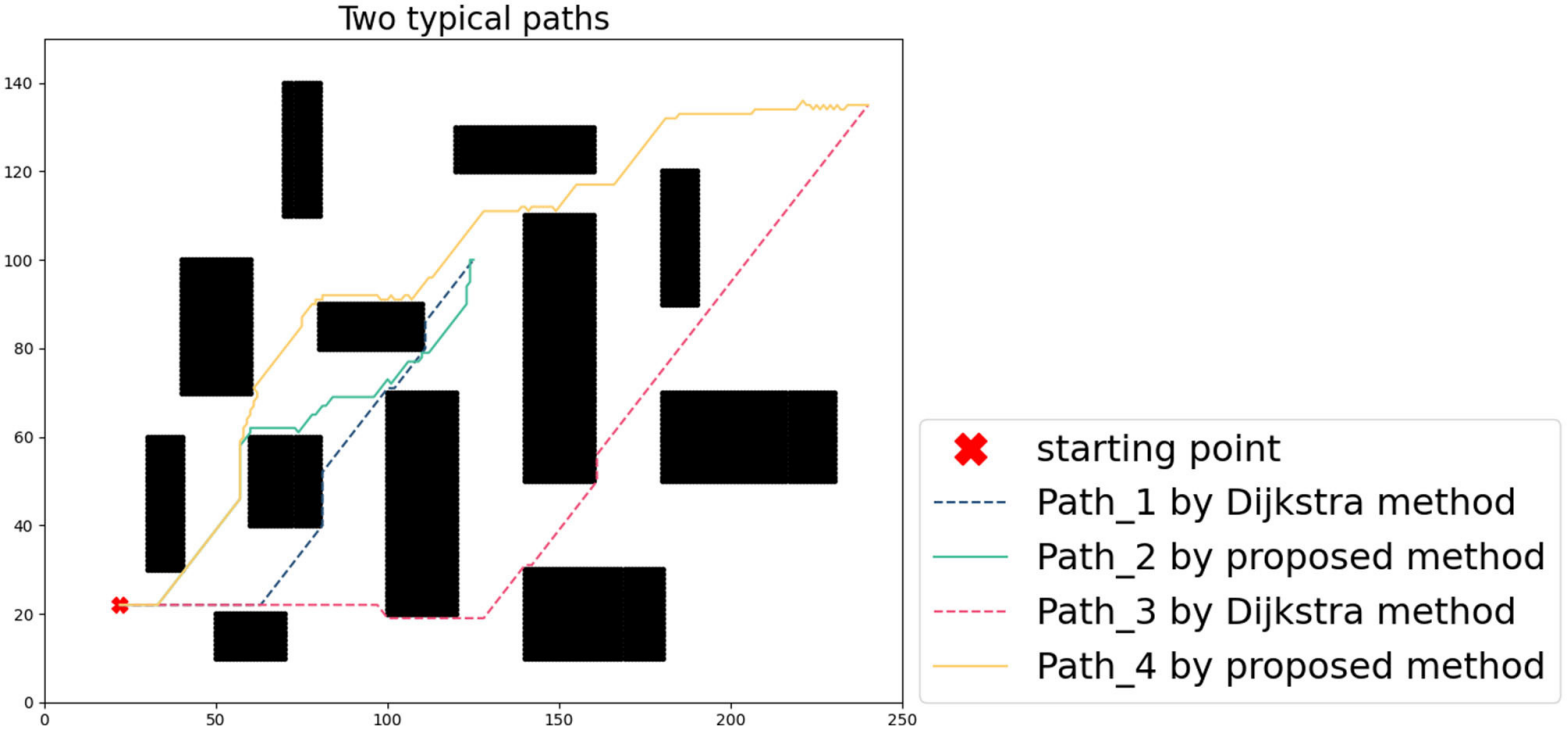
Comparison of two typical paths.

**Figure 7:**
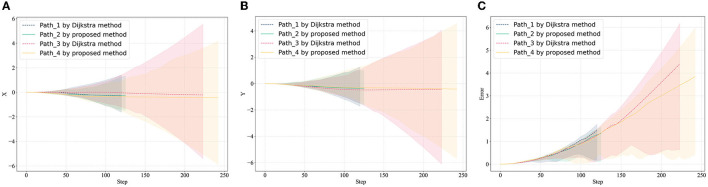
Error estimation in path planning domain. **(A)** Average error in *X*-axis, **(B)** average error in *Y*-axis, and **(C)** average distance error.

Set the starting point as (170, 90), (240, 135), two different paths are obtained through the classic Dijkstra method and the method in this article (the path can also be the same in some scenarios, especially the scene where the path does not pass through obstacles). In Figure 6, the results of different algorithms are represented by dashed and solid lines, respectively. In the case that the noise of the measuring sensor conforms to the Gaussian distribution, the path error calculated by 1,000 Monte Carlo runs is shown in Figure 7. The cumulative error only considers the starting point and the endpoint, and the problem of large error boundaries caused by the path process will not be within the scope of this article.

To reflect the effectiveness of the algorithm, this article selects 8 typical points, and compares the cumulative error statistics of algorithm results and the classical Dijkstra planning results, as shown in Table 1. The proposed algorithm can effectively reduce cumulative error when the sensor is biased. When a robot relies on its inertial navigation, it is easy to deviate from the default path. During the tracking process based on the proposed algorithm path, the endpoint is closer to the target point. The reduction of cumulative error verifies the effectiveness of the proposed algorithm.

**Table 1:** Comparison of cumulative error of different methods for typical endpoints.

**Endpoint**	**Dijkstra method**			**Proposed method**			**Error reduction ratio (%)**
	**x**	**y**	**Error**	**x**	**y**	**Error**	
(170, 90)	−0.07575	−0.41536	2.324235	−0.46782	−0.33134	2.171227	7.0471
(90, 60)	−0.22252	−0.28665	0.621104	−0.17048	−0.28777	0.541869	14.62261
(130, 105)	−0.30649	−0.44412	1.634435	−0.29347	−0.36458	1.485441	10.03032
(175, 140)	−0.28543	−0.38564	2.698455	−0.43593	−0.45941	2.675029	0.875752
(240, 135)	−0.02745	−0.39179	4.497923	−0.37665	−0.36357	3.914085	14.91635
(200, 110)	−0.07465	−0.32521	3.181955	−0.32244	−0.37822	2.857053	11.37192
(125, 100)	−0.28511	−0.4254	1.49236	−0.2927	−0.4051	1.369209	8.994302
(87, 145)	−0.45045	−0.28108	1.596313	−0.49804	−0.25758	1.564632	2.024825

**Figure 8:**
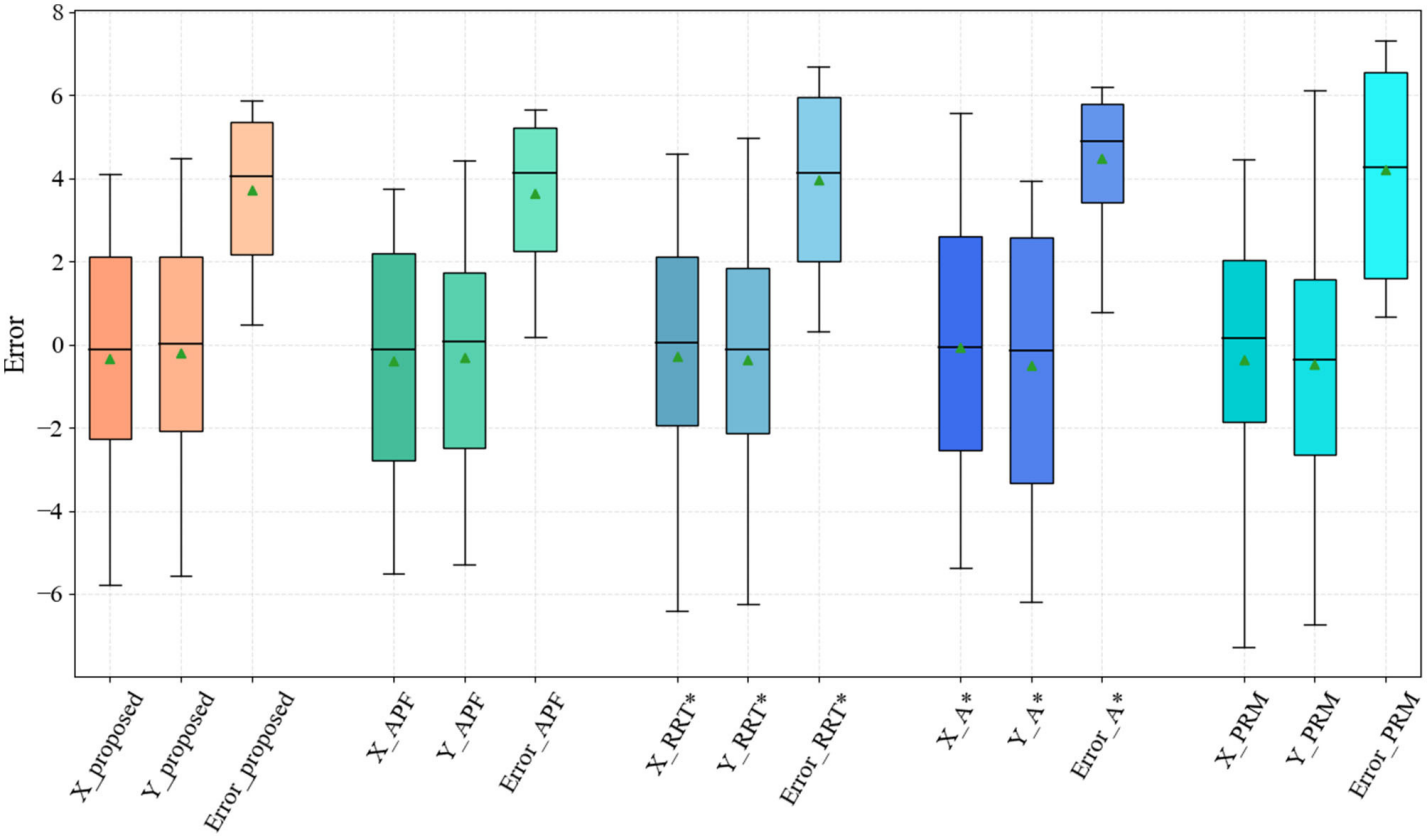
Results of the proposed method compared with artificial potential field (APF), RRT*, A*, and probabilistic roadmap (PRM) methods.

The proposed method is an iterative extension of Dijkstra, and the level of cumulative error in its planning results is significantly improved compared with the original method. To evaluate the effectiveness of the proposed method, the planning results of the proposed algorithm were evaluated in comparison with typical path planning methods, such as the artificial potential field method (APF) (Wang et al., 2020), Grid-based RRT* (RRT*) (Chao et al., 2018), A* (Zafar et al., 2021), and probabilistic roadmap method (PRM) (Agha-mohammadi et al., 2014) methods. The error levels of the different methods were analyzed by bypassing 1,000 Monte Carlo tests under measurement white noise, as shown in Figure 8.

The proposed method has an advantage over the existing probability-based, graph search-based planning methods at the path error level. Note that the APF method falls into local optimum several times in the test, especially in maps containing recessed obstacles, while the method in this article does not have this problem. Among the compared methods, the PRM-based planning method has the largest path accumulation error on account of the probabilistic uncertainty.

## 5. Discussion

The results of the proposed method are the same as those of the Dijkstra method when the path from the starting point does not pass through obstacles, i.e., the shortest path is also the path with the smallest error. The advantage of the proposed planning method is demonstrated after the path encounters and bypasses the obstacles. Unfortunately, the computational effort of the proposed algorithm increases exponentially as the number of obstacles increases. The algorithm in this article is suitable for applications in scenarios with sparse obstacles (e.g., underwater obstacle avoidance for AUVs). The discretization of the map contributes to the lack of smoothness of the planned paths, which can be optimized at a later stage by smoothing algorithms. This will also be a problem that we need to solve in the future. Theoretically, the proposed algorithm achieves pathfinding with minimum estimation error by traversing the global map.

## 6. Conclusion

To address the problem of path planning in the absence of missing global positioning, a path planning algorithm with minimum cumulative error considering sensor drift is proposed. First, the statistical characteristics of sensor noise relative to the cumulative error of the measurement are analyzed. Second, considering the cumulative error in the positioning process, the greedy search algorithm is used to traverse the global map and generate an initial error map. Finally, the proposed algorithm is iterated to generate a smooth global error map, and the path planning task is carried out accordingly. Through simulation analysis and comparison of results, the algorithm significantly improves the safety of collision avoidance during tracking and effectively reduces the cumulative error in complex conditions.

The motion of robots is continuous and regular. Future study will need to accommodate continuous motion strategies and complex path planning tasks in multidimensional spaces and incorporate robot kinematic models to accommodate more types of robots.

## Data Availability Statement

The original contributions presented in the study are included in the article/supplementary material, further inquiries can be directed to the corresponding author.

## Author Contributions

CW provided the original motivation and idea. CC performed the writing of the manuscript and data analysis. DY completed the design of the simulation experiment. GP provided financial support and FZ is responsible for the resources and revision of the manuscript. All authors contributed to the article and approved the submitted version.

## Funding

This study was supported by the National Natural Science Foundation of China (52171322), the National Key Research and Development Program (2020YFB1313200), and the Fundamental Research Funds for the Central Universities (D5000210944).

## Conflict of Interest

The authors declare that the research was conducted in the absence of any commercial or financial relationships that could be construed as a potential conflict of interest.

## Publisher's Note

All claims expressed in this article are solely those of the authors and do not necessarily represent those of their affiliated organizations, or those of the publisher, the editors and the reviewers. Any product that may be evaluated in this article, or claim that may be made by its manufacturer, is not guaranteed or endorsed by the publisher.
